# Effectiveness and safety of Bushen Huoxue granules in treatment of premature ovarian insufficiency: study protocol for a randomized, double-blinded, placebo-controlled, and multicenter clinical trial

**DOI:** 10.1186/s13063-021-05838-w

**Published:** 2021-12-04

**Authors:** Ying Cao, Peijuan-Wang, Yan Lu, Yue Chen, Si Chen, Weibo-Zhao

**Affiliations:** 1 Affiliated Hospital of Integrated Traditional Chinese and Western Medicine, Nanjing University of Chinese Medicine, Nanjing, China; 2 Department of Obstetrics and Gynecology, Affiliated Hospital of Nanjing University of Chinese Medicine, Nanjing, China; 3 Department of Obstetrics and Gynecology, Jiangsu Province Academy of Traditional Chinese Medicine, Nanjing, China

**Keywords:** Chinese herbal medicine, Premature ovarian insufficiency, Bushen Huoxue, Clinical trial protocol

## Abstract

**Introduction:**

Premature ovarian insufficiency (POI) seriously affects the quality of life, endocrine function, and fertility of women of childbearing age. Currently, hormone replacement therapy for POI has some limitations, either with low efficacies or high side effects. Bushen Huoxue (BSHX) plays an important role in alleviating clinical symptoms and improving health status of POI patients. This placebo-controlled, randomized, double-blind, and multicenter clinical trial protocol aims to evaluate the effectiveness and safety of BSHX in women with POI.

**Methods and design:**

We plan to recruit 150 women with POI from four participating hospitals in China. Participants will be randomized in a 1:1 to receive oral BSHX or BSHX placebo. All participants will be treated for 3 months and will be followed up for another 3 month. The primary outcome is questionnaire scores based on the changes in the total symptoms, which is the Chinese version of the Menopause-Specific Quality of Life (CMS) (Nie G, Yang H, Liu J, Zhao C, Wang X, Menopause 24(5):546–554, 2017). CMS will be measured before the intervention, at 3 months and 6 months after randomization for all participants. The other measurements include serum sex hormone levels, anti-Müllerian hormone (AMH) levels, ovarian peak systolic velocity (PSV; cm/s), and antral follicle count (AFC). In this study, the regulatory effects of traditional Chinese medicine on hormones were evaluated by the changes of serum sex hormone levels, which include serum estradiol (E2), luteinizing hormone (LH), and follicle-stimulating hormone (FSH). These indicators will be measured before intervention and at 3 months after randomization.

**Ethics and dissemination:**

This study was approved by the Research Ethics Committee of Jiangsu Province Hospital on Integration of Chinese and Western Medicine (2019LWKY014). All participants will provide written informed consent prior to randomization. The results of this research will be presented to academic conferences and peer-reviewed journals.

**Trial registration:**

ChiCTR1900028451. Registered on 22 December 2019, https://www.chictr.org.cn/index.aspx.

**Supplementary Information:**

The online version contains supplementary material available at 10.1186/s13063-021-05838-w.

## Strengths and limitations of this study

►The results from this multicenter, randomized, double-blinded, placebo-controlled clinical trial will provide new evidence of the efficacy of Bushen Huoxue for premature ovarian insufficiency.

►This is a well-designed study to assess the efficacy and safety of Bushen Huoxue for the treatment Premature Ovarian Insufficiency.

► We use effective and non-invasive methods Chinese version menopause-specific Quality of Life questionnaire to detect the efficacy of drugs, which is easy for investigators to operate and more acceptable to participants.

► Larger-scale clinical studies of Bushen Huoxue among more diverse populations are needed to further confirm these findings.

## Introduction

POI is a clinical syndrome in which ovarian activity declines in women before the age of 40. It is characterized by menstrual disorders (such as menopause, rare menstruation) and accompanied by high gonadotropin and low estrogen, which seriously affects the reproductive health and quality of life of women of childbearing age [1]. In recent years, with the increasing fierce social competition and the change of working style, the incidence of POI among women under 35 years old is about 4%, and it is increasing year by year and showing a younger trend [2]. In addition, there are many causes of iatrogenic POI [3].

The etiology and pathological mechanism of the disease are not yet available, so modern medicine has no effective method to restore ovarian function. The most commonly used treatment is hormone supplementation until the average age of natural menopause in people with no contraindications. Some women need this therapy for decades. But, In 2017, the United States Preventive Services Task Force reviewed the evidence that did not address hormone therapy for preventing or treating menopausal symptoms [4]. In previous clinical studies, the results showed that estrogen replacement therapy did not increase pregnancy rates or follicle formation in SPOI women [5, 6] and had no positive effect on the long-term management of bone mineral density in women with SPOI [7]. More than that, the risk of developing tumors is increased if progesterone and estrogen are used in combination [8]. Furthermore, women who take oral estrogen than transdermal estrogen have an increased relative risk of stroke [9]. If women have a history of breast or ovarian cancer, hormone therapy should be replaced [10, 11].

To solve the current problems in POI treatment, our goal is to search for an effective therapy for POI with fewer side effects. In China, traditional Chinese medicine (TCM) therapy has been widely used to treat POI for thousands of years. According to TCM theory, the basis of female physiology is blood, and her reproductive capacity is dominated by kidney gas. Kidney deficiency and blood stasis are the basic pathogenesis of POI [12]. BSHX is a mixed herbal formula that has been used to treat POI by systematically balancing the body’s Yin and Yang, and correcting the pathophysiological condition of kidney deficiency and blood stasis. Therefore, the BSHX plays a role in the treatment of POI. It is a compound medicine consisting of ten different Chinese herbals (Table S1 in supplementary materials).

Previous clinical studies have shown that BSHX can improve patients’ quality of life by alleviating symptoms, such as lower abdominal pain, hot sweat, lower back, and weak knees. At the same time, BSHX can effectively regulate the level of serum FSH and E2 and improve ovarian function. Therefore, it is worth popularizing and using in clinic [13]. Related animal experiments have proved that BSHX has obvious therapeutic effect on POI mice, which may be through immune protection, reducing the infiltration and damage of ovarian inflammatory cells [14]. Moreover, pharmacological studies have demonstrated that BSHX can interfere with POI through many aspects, which can not only regulate reproductive endocrine hormones, but also prevent premature apoptosis of follicles, improve pelvic blood microcirculation, increase ovarian volume and cavity follicle count, and regulate related signal transduction pathways. Li et al. reviewed the literature on randomized controlled clinical trials of BSHX in the treatment of POI. A meta-analysis of clinical total effective rate, symptom score, menstrual recovery rate, and improvement of serum sex hormones was conducted. The results showed that BSHX appears to be a safe and effective drug to treat patients with POI, and herbal may be superior to western medicines [17]. Studies initially reveal that BSHX interferes with POI through multiple targets. The comprehensive network effect of points, multiple links, and multiple mechanisms have achieved certain research results [15]. However, the study did not include a placebo control group, and there have been few clinical trials with strictly relevant designs. Therefore, in this study, the aim is to conduct a double-blind, randomized, placebo-controlled, multicenter trial of women with POI in China. The primary objective is to evaluate the effectiveness and safety of BSHX treatment for women with POI.

## Methods

### Study design and participants

This is a randomized, double-blinded, placebo-controlled, four-center clinical trial protocol for studying the effects of BSHX on women with POI. There are four hospitals (Table S2 in supplementary materials) that have participated in this project. The clinical trials were conducted according to the principles of the World Medical Association Declaration of Helsinki and the guidelines of Good Clinical Practice of the International Conference on Harmonisation (ICH-GCP). The trial was registered as ChiCTR1900028451 at the Chinese Clinical Trial Registry (https://www.chictr.org.cn/index.aspx) on 22 December 2019. This study has been approved by each hospital’s ethics committee and the responsible regulatory authorities. All participants will provide written informed consent before randomization. Qualification screening and information on demographic and clinical characteristics will also be collected. After passing the qualification screening, participants will undergo a number of medical examination to obtain baseline data. These data are the minimum eligibility criteria for each participant. Meanwhile, it is possible to screen out respondents who may not follow the research procedures. All study participants will be randomly divided into two parallel groups, the BSHX group, and the placebo group, respectively. The trial consisted of 3 months of treatment, interviews at 2-week intervals, and 3 months of follow-up, interviews at 4-week intervals. Based on previous clinical trial studies, we will use a 3-month drug observation period to monitor the safety and efficacy of the treatment [16, 18–20]. The study design flow chart is shown in Fig. S1 (in supplementary materials).

### Sample size calculation

Sample size estimation refers to the calculation of the required sample size in order to meet the accuracy and reliability of statistics (the control of type I errors and the guarantee of inspection efficiency) [21]. The sample size of this study was based on the relevant literature reviews and the validity of hypotheses. The main indicator of this study is to detect the difference of the total score (CMS) between the two groups before and after treatment. There is one randomized clinical trial for BSHX in the treatment of POI [13]. However, there is no placebo control group in the clinical trial and the primary outcome measurements were different. Thus, the parameters are not suitable to estimate the sample size of the trial. We referred to other clinical trials of traditional Chinese medicine in the treatment of POI patients [22]. We assumed that BSHX administration would reduce CMS score by 2.9 points and the SD is 7.42 in the study [23]. If a type I error rate of *α* = 0.05, a power of 90 % (type II error rate of *β* = 0.1), then u1 − *α* =1.64, u1 − *β* =1.28. n1 = n2 ≈ 70. During the 3-month treatment period, taking into account the possible protocol violations, and the maximum possible drop-out rate of 15%, we need 148 participants in total to achieve the number of people required for the efficacy analysis. One hundred fifty participants will be recruited for this study, and the following formula was used to estimate the sample size:

$$ n=2\times {\left[\frac{\left({z}_{1-\raisebox{1ex}{$\partial $}\!\left/ \!\raisebox{-1ex}{$2$}\right.}+{z}_{1-\beta}\right)\sigma }{\delta}\right]}^2 $$

### Patient and public involvement

Patients and the public were not involved in the trial design, and they will not participate in the guidance, or implementation of this study. Once the trial is completed, the results will be disseminated through social media, academic conferences, or research publications.

### Participants and recruitment

There were two ways to recruit patients. One was to display recruitment posters of the study in all participating hospitals. The posters will contain brief descriptions of inclusion and exclusion criteria, the medicines, the ways of participation, and study purposes. Another way is to publish announcements on the hospital’s website. Prior to the start of the trial, all researchers are clinical obstetricians and received formal professional training to do diagnostic interviews. If there were people who were not eligible or refused to participate, we recorded the detailed reasons.

#### Inclusion criteria

Diagnostic criteria are based on the guidelines of the European Society of Human Reproduction and Embryology 2016. Subjects must meet all of the following requirements:

1. Chinese women aged less than 40 years
2. Oligomenorrhea/amenorrhea for more than 4 months
3. At least 2 basal FSH level more than 25 IU/L, on two occasions more than 4 weeks apart
4. Sign the informed consent

#### Exclusion criteria

Excluded individuals who have met either one of the following criteria:

1. Patients with serious diseases, such as malignant tumor and thrombosis
2. Patients with severe mental disorders
3. Congenital abnormal development of reproductive organs, or amenorrhea caused by acquired organic lesions and injuries
4. People with chronic diseases that requires hormone therapy
5. Pregnant or breastfeeding women
6. Patients are taking drugs or acupuncture that could affect menstrual cycle or ovarian function, or had participated in another clinical trial in the previous 3 months that could affect the reproductive system
7. The patient is allergic constitution or allergic to the study drugs
8. Drink or use drugs

### Removal, dropout, suspension, and termination criteria

During the clinical trial, participants who violate the protocol operation will be removed their participation, for instance, failed to take the drug in accordance with the regulations (medication compliance less than 80%) that affect the judgment of drug efficacy because incomplete data will affect the judgment of efficacy and safety. Participants can voluntarily drop out at any time during the trial. Emergency unblinding or loss of eligible subjects to follow-up will be considered dropping out. If the participant drops out, the last recorded data will be recorded and analyzed. These will be recorded in detail in electronic medical records. If participants have any of the following circumstances, the drug treatment discontinues immediately, while safety or post-treatment visits may continue. These circumstances were (1) a serious adverse event occurred, (2) pregnancy, (3) use of medication that can affect menstrual cycle or ovarian function, (4) the participant withdrew the informed consent form, and (5) the subjects showed hypersensitivity reactions to BSHX, such as stomach pain and diarrhea. Once the blindness is made public, or the opening rate of emergency letters exceeds 20% of the sample size, it means that the double-blind experiment will be invalidated and the entire study will be terminated.

### Randomization and blinding

The randomization sequence is generated by a computer and randomization numbers are hidden in sealed, opaque, and sequentially numbered envelopes, which are properly kept until the end of the trial. Participants will be randomly assigned (1:1) using a computer-generated randomization sequence to receive oral BSHX or BSHX placebo. The statistician who is not involved in recruiting will prepare the randomization list, and the list will not be available to anyone in any center. Researchers who do not participate in the recruitment label the envelopes. Meanwhile the size, color, taste, and shape of the placebo are consistent with the corresponding drugs, and all study groups assigned by different numbers will use the same packages of medicines. All the documents related to the randomization and allocation sequence generation will be kept in a box in a locked cabinet away from the clinical environments. Packages of BSHX and placebo were provided to randomly assigned two groups of women in each hospital’s centers. This is a double-blind trial, in which all researchers and subjects will be blinded for the treatment allocation until the trial is completed. If there is a serious adverse event or an emergency unblinding event, the blind will be immediately broken.

### Intervention

All clinics followed the same study protocol. BSHX granules and placebo granules were assigned to different groups for a period of 3 months of treatment. The prescription of placebo is composed of 98% maltodextrin, 2% caramel, and very little bitterant. The BSHX and placebo were manufactured by Jiangyin Tianjiang Pharmaceutical Co. Ltd. (Jiangyin, Jiangsu Province, China) in compliance with China Good Manufacturing Practice standards. Both BSHX and placebo were approved and regulated by the China Food and Drug Administration (FDA) and can be valid for 2 years. The medicines have been widely used in our hospital for many years, and the clinical efficacies were noticeable. Different from other traditional herbs prepared by boiling, it is easier and quicker by dissolving BSHX or placebo (one bag three times a day, 30 g/bag) in water for 30 min. Treatment was taking these medicines after meals three times a day for 3 months. After treatments, medicine packages will be required to return, and compliance will be calculated based on the residual medication. Dosages of Chinese medicines were in accordance with the latest standards of the Chinese Pharmacopoeia (2015 edition). Trial records were confidential and secure.

### Follow-up

All included patients will be re-evaluated at 2, 4, 6, 8, 10, 12, 16, 20, and 24 weeks’ follow-ups. Wherever in-person follow-up was not possible, video conferencing or telephone follow-up was carried out.

### Safety assessments

Studied medicine safety testing includes liver and renal function test, blood routine examination, urine routine examination, stool routine examination, and electrocardiogram. These parameters will be obtained both at screening and after treatment, and all samples were tested by the Jiangsu Province Hospital on Integration of Chinese and Western Medicine in China. Standardized protocols exist at the study center regarding the management of known of potential adverse effects. Adverse events related to the intervention will be monitored through review of the patient’s records, participant self-report, and by the clinicians. All adverse events during treatment and follow-up will be recorded and analyzed in detail. If a participant has a serious adverse event and was not safe to continue the trial, the participants were requested to withdraw from the trial. Immediate relevant treatments were provided to those participants, and following up continued until the response was terminated.

### Outcome measures

#### Primary outcome

The primary outcome measurement was the CMS questionnaires. The CMS was translated by Yang Hongyan and others [24]. In clinical practice, the Chinese version of the MENQOL Questionnaire (Table S3 in supplementary materials) has been proven to be highly reliable, effective, and responsive. It is a reasonable and effective tool to comprehensively evaluate the current physical and mental health problems of menopausal women in China [25]. CMS has a total of 29 items, which are divided into 4 dimensions, including vasomotor symptoms (3 items), psychosocial symptoms (7 items), physiological health (16 items), sexual health-related questions (3 items). Differences in this questionnaire are that MENQOL appears not only as symptoms based on the frequency, it also includes the troubles caused by various severity of symptoms degree. Participants will complete the questionnaires before, after the intervention, and follow-up. Items measured and data collection schedule are shown in Table S4 (in supplementary materials).

#### Secondary outcomes

The secondary measurements included serum sex hormone levels, AMH, AFC, and PSV (cm/s). The regulatory effect of tested medicine on hormone balance was monitored by the level of serum sex hormones (FSH, LH, E2). The blood samples of all subjects were taken in the early antral follicular phase before treatment and after the withdrawal of the drug. As an ovario specific growth factor, AMH is an effective and reliable indicator to predict ovarian reserve function [26], and it has no cycle-dependent [27]. AFC is helpful to evaluate the relative accuracy of ovarian reserve function when used in combination with sex hormone detection and can also provide a reference for the formulation of the treatment plan [28]. Transvaginal ultrasound is the best way to observe AFC indicators, which can be accurately evaluated and calculated the number of follicles. Generally, the AFC is measured at the beginning with the early antral follicular stage. Transvaginal ultrasound is performed by an experienced ultrasound expert, and the follicle count of both ovaries with a diameter of 2–10 mm (measured by clinicians rather than automatically calculated) is used to calculate AFC. All subjects underwent vaginal ultrasound examination at the follicular stage before treatment and after drug withdrawal.

### Quality control

The multicenter clinical trial is a complex process, and quality control is a reliable guarantee for this trial. Effective quality control measures were carried out to ensure the quality of the trial and the accuracy of results, completeness, and authenticity of the information. At the same time, the rights and welfare of participants should be guaranteed. Being the authority among research centers, Jiangsu Province Hospital on Integration of Chinese and Western Medicine is in charge of formulating the standard operating procedures (SOPs) training, and visiting each site regularly to guidance the trial, which strictly followed the protocol. The data were collected timely, directly, accurately, and clearly. The recordings were regularly self-checked its accuracy and completeness. Any errors were corrected in accordance with the prescribed methods. Data from case reports were published on the public clinical trial management platform (www.medresman.org) within 6 months after the end of the trial. An independent data safety monitoring committee, which is independent of the sponsor without competing interests, was in charge of data validation.

### Statistical analysis

Data will be analyzed with SPSS V.21 (IBM) by statisticians independent from involved researchers in this study. Efficacy and safety will be evaluated by intention-to-treat (ITT) analysis within the full analysis set (FAS) according to the ITT principle. At the same time, per-protocol (PP) analysis will apply to evaluate data collected from subjects who have completed all steps of the trial protocol. Results of the full analysis set and the per-protocol set will be compared. All prespecified outcomes will analyze in the ITT population because ITT analysis often underestimates the efficacy and was usually conservative. On the contrary, PP analysis overestimates the efficacy and the differences between the two groups. Therefore, this study mainly uses ITT analysis, and PP analysis will be applied if there was a statistical significance. Various assignment methods were used to detect whether the results of different assumptions of missing data are robust. The safety evaluation will be conducted by ITT analysis.

Descriptive analysis will be used for baseline variables including demographic characteristics, medical history, course of disease, treatment history, combined diseases, concomitant medications, vital signs, laboratory tests for safety assessment, and adverse events. Continuous variables will be expressed by mean ± SD; categorical variables will be shown as counts and percentages. Estimates of the adjusted differences in risks are presented with 95% confidence intervals (CIs) of the difference. The comparability of the characteristics between the BSHX and placebo groups will be assessed by using *t*-test for continuous variables with normal distribution, while the non-parametric Mann-Whitney-Wilcoxon test will be used for the comparison of data with non-normal distribution. Specifically, the paired *t*-test will be used to compare the difference of the outcome between preintervention and postintervention in each group and independent t-test will be used to compare the difference between the two groups. Categorical variables will be compared using *Χ*^2^ statistics, while the Fisher exact test will be used when the theoretical frequency is less than 5 in more than 25% of the cells. In order to control the center and baseline effects, covariance analysis will be applied for the intergroup comparison with continuous variables and Cochran-Man-tel-Haenszel test for categorical variables. The differences of CMS scores, levels of FSH, LH, E2, AMH, the AFC, and ovarian PSV (cm/s) at month 0 and month 3 between the BSHX and placebo groups will be compared using an independent *t*-test. A repeated-measures analysis of variance (ANOVA) test with Bonferroni post hoc test will be used to evaluate the changes in CMS scores, levels of FSH, LH, E2, AMH, the AFC, and ovarian PSV (cm/s) and safety assessment measurements throughout the experiment. In all tests, a value of *p* < 0.05 will be considered statistically significant. All statistical analyses will be performed using SPSS V.20.0.

## Ethics and dissemination

The protocol has been approved by the Ethics Committee of Jiangsu Province Hospital on Integration of Chinese and Western Medicine. (No. 2019LWKY014). It has been registered in the Chinese Clinical Trial Registry (ChiCTR1900028451). The data of this trial will be managed by ResMan at http://www. medresman. org/ and posted on Chinese Clinical Trial Registry. Results from this study will be published to the public through social media, academic conferences, and peer-reviewed journals.

## Discussion

To our knowledge, this is the first study protocol of a randomized, double-blinded, placebo-controlled, multicenter clinical trial for POI treatment. The results from this trial will provide reliable evidence for the application of Chinese medicine BSHX in the treatment of POI. Report of this randomized controlled trial has been complied according to the Consolidated Standards of Reporting Trials for TCM statement [29]. This clinical trial protocol followed quality methodology and strictly enforced quality control. A standardized preparation of traditional Chinese medicine particles, record screening, randomization process, verification of biomarkers, and objective measurement were formulated by this study. Detailed methods of distribution hiding, recruitment, randomization, and data collection were also obtained. Based on previous studies, we have concluded that the most important clinical efficacy evaluation method of Chinese herbal medicine is the effect of Chinese herbal medicine on specific symptoms of diseases. Symptoms last for several years in many POI cases, and this seriously affects the quality of life of patients. Therefore, it is imperative and necessary to search for medicines that can significantly alleviate symptoms with no adverse reactions in the long term. Results from previous clinical trials have shown that BSHX can effectively relieve clinical symptoms, regulate hormone levels, and restore menstrual cycle and menstrual flow [30]. Other studies also demonstrated BSHX has the effect of improving the luteal function, improving the pregnancy rate, and enhancing the body’s immunity [31]. It has good clinical effect and is an effective prescription. Modern pharmacological studies suggest that Chinese herb medicines in BSHX can effectively regulate female hypothalamus-pituitary-ovarian reproductive axis and promote follicle growth and maturity, with strong estrogen-like action. For example, Cuscuta, one of the Chinese herb medicines in BSHX, enhances the function of human chorionic gonadotropin (HCG) and LH receptors of ovary and pituitary to release gonadotropin release hormone (LRH) reactivity [32]. Epimedium can directly act on the hypothalamus and promote the pituitary releasing hormone and has a gonadal-like effect with direct adjustment function [33]. Previous papers indicated that blood activating drugs can improve ovarian reactivity, inhibit the activity of anti-ovarian antibodies, and reduce the ovarian autoimmune damage by improving ovarian blood supply [34]. Results from the abovementioned studies are obvious evidences that BSHX is effective and safe for POI treatment. The main purpose of this study is to explore the factors of diagnosis or treatment rather than pathogenesis. It is, therefore, important to further investigate the underlying mechanism of BSHX in POI treatment in humans, such as exploration of POI-related signal pathway regulation, the requirements of POI disease itself. These studies in the future are expected to open up new ideas for the treatment of POI.

## Trial status

The protocol version number is 1.3, dated January 10, 2020. After getting the ethical approval, there have been minor protocol modifications because of the COVID-19 pandemic as described above, which was communicated at the time with involved wards. And re-report to the ethics committee in a timely manner. These changes have no impact on the study conduct. Recruitment began in February 2020 and is expected to complete patient follow-up by the end of November 2021.

## Protocol amendments

Any protocol amendments will be communicated to the sponsor (Jiangsu Province Hospital on Integration of Chinese and Western Medicine), the relevant research ethics committee, and the study participants.

## Supplementary Information

**Additional file 1.** Supplementary Table S1–S4. Supplementary Fig. S1.

## Data Availability

We declared that materials described in the manuscript, including all relevant raw data, will be freely available to any scientist wishing to use them for noncommercial purposes without breaching participant confidentiality. Access to the protocol and the dataset may be provided upon request to the authors.
