# Supplementary material for: Effectiveness and safety of Bushen Huoxue granules in treatment of premature ovarian insufficiency: study protocol for a randomized, double-blinded, placebo-controlled, and multicenter clinical trial
Source: Trials. 2021 Dec 4;22:877. doi: 10.1186/s13063-021-05838-w (PMC8643188; doi:10.1186/s13063-021-05838-w)
Supplement: Supplementary file 1 — Additional file 1. Supplementary Table S1–S4. Supplementary Fig. S1. [file 13063_2021_5838_MOESM1_ESM.docx]

| **Supplementary Material Table 1 Details of Bushen Huoxue** | | | | | |
| --- | --- | --- | --- | --- | --- |
| Chinese name | English name | Ladin name | Source | Pharmacological | Effects weight(%)* |
| TU SI ZI | Dodder Seed | Cuscuta chinensis Lam | Mature seed | Treatment of impotence, seminal emission, dripping of urine after urination, enuresis,blurred vision and tinnitus, threatened abortion due to hypofunction of the kidney | 11.11 |
| CHUAN XIONG | Chuanxiong (Wallich Ligusticum) Equivalent plant | Ligusticum chuanxiong | Rhizome | To move qi and quicken blood, dispel wind and relieve pain | 8.89 |
| SHU DI HUANG | Adhesive Rehmannia Cocked Root | Rehmannia glutinosa | Steamed and sundried root | To supplement blood and enrich yin, boost essence and replenish marrow. | 11.11 |
| ZHI MU | Liliaceae | Anemarrhena asphodeloides Bge. | Dried roots rhizome | To clear heat and drain fire, enrich yin and moisten dryness, eliminate vexation and allay thirst. | 8.89 |
| BAI SHAO | Common Peony | Paeonia albiflora | Root | To calm liver and relieve pain, nourish blood and regulate menstruation, constrain yin and check sweating. | 8.89 |
| CHI SHAO | Red Peony root | Radix Paeoniae Rubra | Dry root | Treatment of maculation in epidemic diseases, spitting of blood, epistaxis, inflammation of the eye, pain in the chest and costal regions, amenorrhea, dysmenorrhea, mass formation in the abdomen, traumatic injuries, boils and sores. | 8.89 |
| DANG GUI | Chinese Angelica Equivalent plant: Phlojodicarpus sibiricus | Angelica sinensis | Root | To nourish blood and regulate menstruation, quicken blood, relieve pain, moisten intestines and relieve constipation. | 11.11 |
| HUANG BAI | Amur Corktree Equivalent plant: Phellodendron chinense | Phellodendron amurense | Bark | To drain fire, dispel damp and resolve toxin. | 11.11 |
| CHAI HU | Chinese Thorowax Equivalent plant: Bupleurum scorzonerifolium | Bupleurum chinense | Root | To harmonize exterior and interior, soothe liver and upbear yang. | 8.89 |
| YIN YANG HUO | Shorthorned Epimedium Equivalent plant: Epimedium sagittatum | Epimedium brevicornu Maxim. | Aerial parts | To supplement kidney and invigorate yang , strengthen sinews and bones, dispel wind-damp. | 11.11 |

*The weight of every single herb in each bag of BSHX (30 g).

| **Table 2 The hospitals participating in this study** | |
| --- | --- |
| Code | Participating hospitals |
| 01 | Jiangsu Province Hospital on Integration of Chinese and Western Medicine |
| 02 | Suzhou Hospital of Traditional Chinese Medicine |
| 03 | Taizhou Integrated and Western Medicine Hospital |
| 04 | Wuxi City Traditional Chinese Medicine Hospital. |

| **Table 3 Chinese Version of the Menopause-Specific Quality of Life Questionnaire** | |
| --- | --- |
| In the last month, have you experienced the symptoms in the following questionnaire? If you have not experienced these symptoms, please tick “√” in the“□” above “No”; if you have experienced this symptom, tick “√” in the “□”above “Yes”, and select a level from "0~6" according to the degree of the symptom affecting you. "0" means not affected at all, and "6" means extremely affected; at 0 Between and 6, the closer to 0, the less affected, and the closer to 6, the greater the impact. | |
| 1.Hot flushes | □ □ → 0 1 2 3 4 5 6  No Yes |
| 2.Night sweats | □ □ → 0 1 2 3 4 5 6  No Yes |
| 3.Sweating | □ □ → 0 1 2 3 4 5 6  No Yes |
| 4.Dissatisfaction with my personal life | □ □ → 0 1 2 3 4 5 6  No Yes |
| 5.Feeling anxious or nervous | □ □ → 0 1 2 3 4 5 6  No Yes |
| 6.Poor memory | □ □ → 0 1 2 3 4 5 6  No Yes |
| 7.Accomplishing less than I used to | □ □ → 0 1 2 3 4 5 6  No Yes |
| 8.Feeling depressed, down or blue | □ □ → 0 1 2 3 4 5 6  No Yes |
| 9.Being impatient with other people | □ □ → 0 1 2 3 4 5 6  No Yes |
| 10.I always want to be alone | □ □ → 0 1 2 3 4 5 6  No Yes |
| 11.Gastric flatulence or distending pain (with farting or belching) | □ □ → 0 1 2 3 4 5 6  No Yes |
| 12.Aching in muscles and joints | □ □ → 0 1 2 3 4 5 6  No Yes |
| 13.Feeling tired or worn out | □ □ → 0 1 2 3 4 5 6  No Yes |
| 14.Difficulty sleeping | □ □ → 0 1 2 3 4 5 6  No Yes |
| 15.Aches in back of the neck or head | □ □ → 0 1 2 3 4 5 6  No Yes |
| 16.Decrease in physical strength | □ □ → 0 1 2 3 4 5 6  No Yes |
| 17.Decrease in stamina | □ □ → 0 1 2 3 4 5 6  No Yes |
| 18.Lack of energy | □ □ → 0 1 2 3 4 5 6  No Yes |
| 19.Dry skin | □ □ → 0 1 2 3 4 5 6  No Yes |
| 20.Weight gain | □ □ → 0 1 2 3 4 5 6  No Yes |
| 21.Facial hair increased | □ □ → 0 1 2 3 4 5 6  No Yes |
| 22.Changes in appearance, skin texture or complexion | □ □ → 0 1 2 3 4 5 6  No Yes |
| 23.Feel uncomfortable with swelling | □ □ → 0 1 2 3 4 5 6  No Yes |
| 24.Low backache | □ □ → 0 1 2 3 4 5 6  No Yes |
| 25.Frequent urination | □ □ → 0 1 2 3 4 5 6  No Yes |
| 26.Involuntary urination when laughing or coughing | □ □ → 0 1 2 3 4 5 6  No Yes |
| 27.Decrease in my sexual desire | □ □ → 0 1 2 3 4 5 6  No Yes |
| 28.Vaginal dryness | □ □ → 0 1 2 3 4 5 6  No Yes |
| 29.Avoiding intimacy | □ □ → 0 1 2 3 4 5 6  No Yes |

| Table4 Schedule of data collection | | | | | |  | | |
| --- | --- | --- | --- | --- | --- | --- | --- | --- |
| Items | Screening period | Treatment period (1-3 months) | | | | Follow-up period  (4-6 months) | | |
| Months | √ | 0 | 1 | 2 | 3 | 4 | 5 | 6 |
| Eligibility screening and signed informed consent | √ |  |  |  |  |  |  |  |
| Inclusion/exclusion criteria | √ |  |  |  |  |  |  |  |
| Demographic characteristics | √ |  |  |  |  |  |  |  |
| Medical history, course of disease, treatment history | √ |  |  |  |  |  |  |  |
| Combined diseases | √ |  |  |  |  |  |  |  |
| Concomitant medications | √ | √ | √ | √ | √ |  |  |  |
| CMS |  | √ |  |  | √ |  |  | √ |
| AFC, PSV |  | √ |  |  | √ |  |  |  |
| Serum sex hormone levels, AMH |  | √ |  |  | √ |  |  |  |
| Vital signs | √ | √ | √ | √ | √ | √ | √ | √ |
| Laboratory tests for safety assessment | √ |  |  |  | √ |  |  |  |
| Adverse events |  |  | √ | √ | √ | √ | √ | √ |

1.CMS: Chinese version of the Menopause-Specific Quality of Life questionnaire. 2.AFC: antral follicle count. 3. AMH: Anti-mullerian hormone. 4.PSV: ovarian peak systolic velocity (cm/s) 5. Safety assessments: Liver and kidney function test, routine blood test, routine urine test, routine stool test and electrocardiogram. 6.Vital signs: temperature, heart rates, breathing and blood pressure.


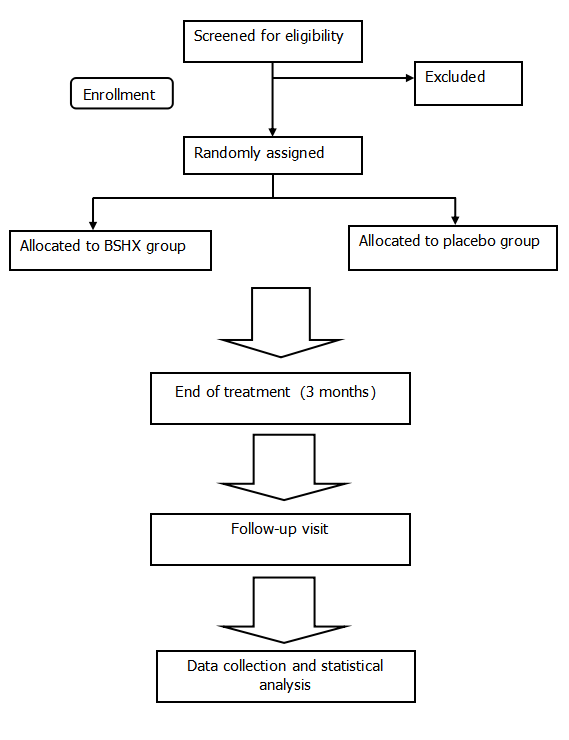


Figure 1 Study design flowchart; POI, Premature Ovarian Insufficiency, BSHX, Bushen Huoxue.
